# The Effect of Strain Rate on Hydrogen-Assisted Deformation Behavior and Microstructure in AISI 316L Austenitic Stainless Steel

**DOI:** 10.3390/ma16082983

**Published:** 2023-04-09

**Authors:** Elena Astafurova, Anastasiya Fortuna, Evgenii Melnikov, Sergey Astafurov

**Affiliations:** 1 Institute of Strength Physics and Materials Science, Siberian Branch of the Russian Academy of Sciences, 634055 Tomsk, Russia; melnikov.evgenii@ispms.ru (E.M.); svastafurov@ispms.ru (S.A.); 2 Department of Physical Materials Science, National University of Science and Technology MISiS, 119049 Moscow, Russia; anastasya_fortuna@mail.ru

**Keywords:** austenitic stainless steel, hydrogen embrittlement, strain rate, strain hardening, microstructure, fracture

## Abstract

The influence of strain rate in the interval of (10^−5^–10^−3^) 1/s on room temperature tensile behavior, dislocation arrangement, deformation mechanisms, and fracture of austenitic stainless steel AISI 316L electrochemically charged with hydrogen was investigated. Independently on strain rate, hydrogen charging provides the increase in the yield strength of the specimens due to a solid solution hardening of austenite, but it slightly influences deformation behavior and strain hardening of the steel. Simultaneously, hydrogen charging assists surface embrittlement of the specimens during straining and reduces an elongation to failure, which both are strain rate-dependent parameters. Hydrogen embrittlement index decreases with increase in strain rate, which testifies the importance of hydrogen transport with dislocations during plastic deformation. The stress–relaxation tests directly confirm the hydrogen-enhanced increase in the dislocation dynamics at low strain rates. The interaction of the hydrogen atoms with dislocations and hydrogen-associated plastic flow are discussed.

## 1. Introduction

Austenitic stainless steels are a very important class of materials for medicine, chemical industry, and energetics [1]. Containers for storage and transportation of liquified hydrogen are often made of austenitic stainless steels. They are susceptible to embrittlement in a hydrogen environment, as are many other metals and alloys, but stable austenitic stainless steels are more resistant against it in comparison with ferritic, dual-phase, or metastable austenitic stainless steels [1,2].

To explain hydrogen embrittlement (HE) on a microscopic level, different mechanisms were suggested. 

(1) Hydride-associated HE is observed in materials where hydrides are brittle and stable during the experiment. However, hydrides do not usually form in austenitic stainless steels [3]. 

(2) Hydrogen-enhanced decohesion (HEDE) mechanism assumes hydrogen-assisted weakening of the interatomic bonds [3,4]. For transgranular cracking, hydrogen should be concentrated in crack tips or in the lattice near the cracks. In this case, high elastic stresses and, consequently, high hydrostatic pressures are needed to produce decohesion. Moreover, V. Gavriljuk [5] showed that hydrogen forces the metallic character of the bonds in austenite, which assists plasticity of the steel. In the case of intergranular fracture, grain boundaries work as traps for hydrogen atoms [3]. However, C.J. McMahon points out that there is no evidence of hydrogen transport to the grain boundaries in concentrations large enough to cause fracture of the steel [6].

(3) P. Rozenak associated HE in austenitic stainless steel AISI 304 with the formation of α’-martensite [7]. Later, T. Michler at al. [8] showed that embrittlement can occur in the absence of the martensitic phase in stable austenitic steels. 

(4) Hydrogen-enhanced localized plasticity (HELP-theory), promoted by Birnbaum [9], is the most common theory nowadays. According to the HELP theory, interaction between hydrogen atmospheres and dislocations is responsible for the HE. Solute hydrogen atoms reduce the repulsive forces between dislocations of one sign due to the shielding of their elastic fields. This effect leads to the coalescence of dislocations and, as a result, to the formation of the microvoids and cracks. In situ TEM observation of the dislocations’ motion in pure aluminum conducted by P.J Ferreira, I.M. Robertson, and H.K. Birnbaum [10] completed this theory with data about the hydrogen-assisted decrease in the staking fault energy (SFE) and stabilization of the edge components of dislocations, which make dislocation structure planar and cause slip localization. According to the theory, cracks could propagate both through grains bodies and along boundaries depending on the location of hydrogen [3].

Although austenitic stainless steel is a relatively studied object, the HE effects are sometimes contradictory in this class of steel, and the individual influence of different factors should be explored in detail to generalize HE in them. In particular, D.P. Abraham and C.J. Altstetter [11] showed that hydrogen charging increases the yield strength of austenitic steel AISI 310 but does not change its total elongation. However, for single crystals of AISI 316 steel, M. Koyama et al. [12] found out that hydrogen charging lowers an ultimate tensile strength (UTS) but does not have a significant effect on its yield strength. For high-interstitial fully austenitic 0.6C-12Mn Hadfield steel and 1C-31Mn-9Al steel, a hydrogen-assisted softening, noticeable decrease in UTS, and reduction in elongation at rupture was observed; for 18Cr-19Mn-0.8N and 0.6C-23Mn TWIP steels, a hardening and significant fall in elongation at rupture were reported [8]. T. Michler at al. [8], in their report, tried to generalize some important characteristics of HE in stable austenitic steels with different SFEs. They noticed that phase stability does not individually provide the resistance of the steels against HE and other characteristics, such as the deformation mechanism and dislocation arrangement, should be considered. Along with variations in deformation mechanism and dislocation slip mode, the refinement of grain structure with the low-angle boundaries of dislocation nature reduces the HE in 18Cr-8Ni steel, in which deformation-induced phase transformation typically assists it [13]. Consequently, further investigation of the mechanisms of the interaction between solute hydrogen and dislocations in austenitic steel are relevant. In coarse-grained material, the hydrogen–dislocation interaction is possible only at relatively low strain rates, otherwise hydrogen atmospheres would not be able to migrate with moving dislocations [14,15,16]. The importance of the “dislocation-hydrogen’’ interaction during the plastic deformation of stable austenitic stainless steels of 300-series could be confirmed in stress–relaxation and strain rate variation tests.

The aim of this work was to explore the influence of the strain rate on mechanical properties, dislocation arrangement, mechanisms of deformation, and fracture of stable austenitic stainless steel AISI 316L and to evidence the “dislocation–hydrogen” interaction on these parameters.

## 2. Materials and Methods

The chemical composition of the AISI 316L steel is presented in Table 1. The billets with the linear dimensions 20 × 20 × 40 (mm) were cut from the industrially produced steel bars (hot-rolled) using electro-spark unit. Steel billets were solution-treated at 1100 °C for 1 h and quenched into water of room temperature. After solution treatment, steel had an austenitic structure with an average grain size of 19 μm, excluding twin boundaries. Solution-treated materials were not textured.

Tensile specimens were cut from the solution-treated billets in the form of dumbbells with a gage section of 8 × 2.7 × 0.5 (mm). Before hydrogen charging and tension, specimens were mechanically grinded using sanding paper of different dispersion (the direction of the grinding was change to perpendicular one on each sanding paper type). After mechanical grinding, an electrolytic polishing of the specimens in a supersaturated solution of chromium anhydride (CrO_3_) in phosphoric acid (H_3_PO_4_) was performed (U = 15–17 V) until the mirror-like surface was obtained.

**Figure 1:**
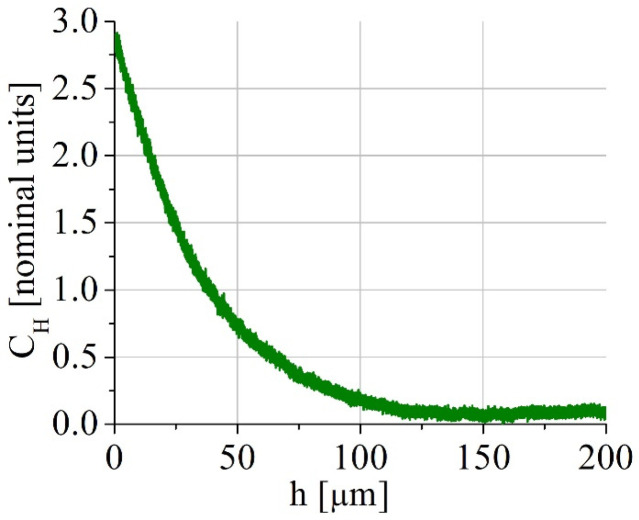
In-depth distribution of hydrogen in the specimen after hydrogen charging for 100 h. The distribution corresponds to the one side surface of the specimen, the opposite surfaces were charged identically.

Hydrogen charging was conducted in 1N H_2_SO_4_ solution (with a CS(NH_2_)_2_ as a recombination potion) at the current density of 50 mA/cm^2^ for 8 and 100 h. Even after hydrogen charging for 100 h, hydrogen was distributed inhomogeneously, as shown in Figure 1, for one side of the specimen (spectrometer GD-Profiler 2, Horiba, France). Each tensile specimen was put into the electrolytic bath between two plane electrodes, so both side surfaces were charged identically. As a result, about the half of the specimens’ width contains hydrogen after hydrogen charging. Such gradient distribution of hydrogen is typical of the electrochemical hydrogen saturation in the millimeter scale specimens, and it models the real industrial saturation of the material, for instance, under cathodic protection of the steel constructions or corrosion [17]. Before tensile tests, hydrogen-charged specimens were kept in liquid nitrogen.

The uniaxial static tensile tests were carried out at ambient temperature using hydrogen-charged and hydrogen-free (initial) specimens. In the study, a universal testing machine LFM-125 (Walter+Bai AG, Löhningen, Switzerland) was employed. During the test, the load (P) and displacement of the moving traverse (Δl = l–l_0_, where l is a length of the deformed sample, l_0_ is an initial length of the sample) were collected. Then, these values were converted to an engineering stress (σ_e_ = P/S_0_, S_0_ is the initial cross-sectional area of a sample) and an engineering strain (ε_e_ = Δl/l_0_). The true stress (σ = σ_e_ (1 + ε_e_))–true strain (ε = ln(l/l_0_)) plots were reconstructed assuming a uniform deformation of a sample at strains lower than that corresponded to an ultimate tensile stress. Using true diagrams, a strain hardening rate SHR = dσ_t_/dε_t_ was calculated. The first part of the tensile tests was conducted with a relative strain rate of 1 × 10^−4^ 1/s till fracture. Hydrogen-free and hydrogen-charged for 100 h (for comparison, specimens charged for 8 h were also used) specimens were explored to investigate the hydrogen-affected microstructure of the steel by transmission electron microscopical (TEM, JEM-2100, JEOL, Tokyo Boeki Ltd., Tokyo, Japan) methods. For TEM study, a part of the specimens was unloaded at 10% strain and mechanically grinded to the thickness of 0.2 mm. Thinning was performed from the one side of the specimen to save the surface hydrogen-affected layer. Flat discs were cut from these thin foils and subjected to electropolishing in a solution of 95% glacial acetic acid (CH_3_COOH) + 5% perchloric acid (H_3_ClO_4_). The electrolyte was sprayed from the grinded side to obtain the TEM observation area in the surface layer, which was saturated by hydrogen and deformed. The other side of the foil was protected with nail polish, which then was removed by acetone. Therefore, all TEM images were obtained in surface hydrogen-affected layers. Indexing of selected area electron diffraction patterns was performed as detailed elsewhere [18].

For hydrogen-free and hydrogen-charged specimens, the stress–relaxation tests were also employed. They consisted of tension of the specimens with the strain rate of 1 × 10^−4^ 1/s up to 10% of plastic deformation, then active traverse was stopped for 60 s or 1000 s without termination of a stress–strain diagram recording. Specimens unloaded after stress–relaxation tests were also studied by the TEM-method. Three specimens were used for stress–relaxation tests in hydrogen-free and hydrogen-charged states.

To investigate the influence of strain rate on the mechanisms of HE, the specimens were tensile tested with the strain rates of 6 × 10^−5^ 1/s, 6 × 10^−4^ 1/s, and 6 × 10^−3^ 1/s. Using TEM, the dislocation arrangements of the specimens, which were unloaded at 5% strain, were also explored. Scalar dislocation density was calculated by standard methods using TEM images [18]. Five specimens were used for each strain rate test, the specimens for TEM study were tested additionally to those used for statistics.

The sides and fracture surfaces of the specimens were studied using a scanning electron microscope VEGA3 (TESCAN, Brno, Czeck Republic).

## 3. Results

### 3.1. Mechanical Tests

#### 3.1.1. Tension at Strain Rate 1 × 10^−4^ 1/s

**Figure 2:**
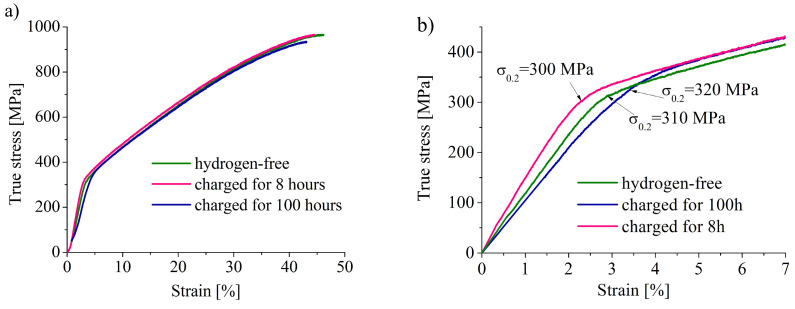

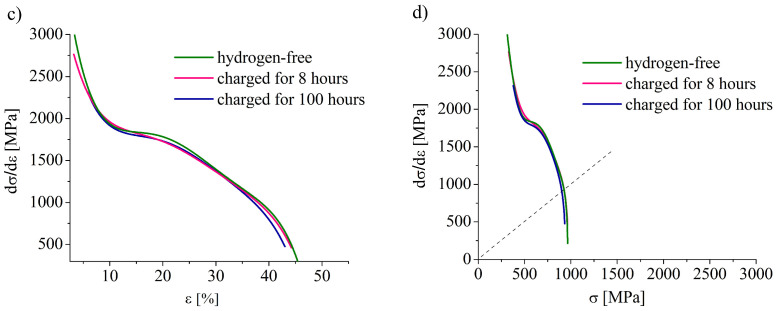
(**a**) True stress–true strain diagrams (only stages of uniform deformation are shown); (**b**) enlarged parts of the diagrams demonstrating the effect of hydrogen charging on the yeild stress σ^YS^ values; and (**c**,**d**) strain hardening coefficient dσ/dε in dependence on true strain and true stress, respectively (dotted line on (**d**) corresponds to Considere’s criterion dσ/dε = σ).

In Figure 2a, the true stress (σ)–true strain (ε) diagrams for hydrogen-free and hydrogen-charged specimens are shown. Hydrogen charging does not influence the stages of plastic flow. The yield strength (σ^YS^) of the hydrogen-charged specimens is higher than that for hydrogen-free one (Figure 2b, Table 2). The ultimate tensile strength (σ^UTS^), total elongation (δ), and uniform strain (ε) all have close values for hydrogen-charged and hydrogen-free specimens, but hydrogen slightly decreases these values. The hydrogen embrittlement index K_H_ = (δ_H_ − δ_0_)/δ_0_, which characterizes the hydrogen-assisted decrease in the elongation of specimens in percent, is dependent on charging duration: 5% after 8 h-charging and 12% after 100 h-charging (Table 2).

The dependences of strain hardening coefficient dσ/dε on true strain and true stress are shown in Figure 2c,d. The dσ/dε value continuously decreases to the strain about 45%. Both types of the specimens do not satisfy a Considere’s criterion (Figure 2d), in other words, the neck on the samples was formed at stress σ > dσ/dε. This is peculiar for materials with planar slip, in which neck formation is difficult due to suppression of a multiple slip [19].

#### 3.1.2. Stress–Relaxation Test

**Figure 3:**
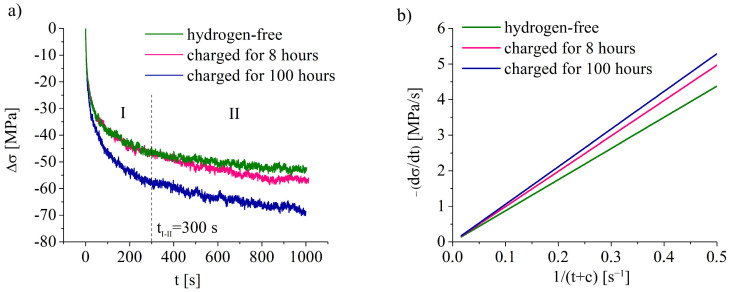
(**a**) The variation in the true stress (stress drop Δσ) during relaxation test vs. relaxation duration (t); (**b**) the first derivative of the true stress in the stage of stress relaxation vs. 1/(*t* + *c*).

Figure 3a presents the dependence of a stress drop due to stress–relaxation (Δσ) on t-value. The stages of the diagrams in Figure 3a are similar for all specimens, but the higher hydrogen charging exposure gives higher stress drop during relaxation test (Figure 3a, Table 3). Before termination of the active tension, specimens were deformed up to the 10% strain. Stresses, corresponded to the relaxation start, are close for hydrogen-free and hydrogen-charged specimens (Table 3). As the process of stress–relaxation is associated with the dislocation movement and annihilation, diagrams in Figure 3a can be used for analysis of the hydrogen–dislocation interaction assuming the only effect of hydrogen charging.

The diagrams in Figure 3a could be divided into two parts (stages I and II), as shown on Figure 3a. The first stage was approximated by a logarithmic function, which is equivalent to the function suggested by D.P. Abraham and C.J. Altstetter [20]:

(1) σ=a−bln(t+c)

where *σ* is true stress, *t* is the duration of the stress–relaxation test, *a*, *b,* and *c* are constants. In order to find the rate of the stress–relaxation on the stage I, the function (1) was differentiated by a (*t + c*) variable. Figure 3b presents the relation between the first derivative of the true stress with respect to a 1/(*t* + *c*) variable (*c* ≈ 1), as it was made in the paper [20]. According to this analysis, the rate of stress–relaxation was the lowest for the hydrogen-free specimen and this rate grew with hydrogen charging (Figure 3b).

The stage II, which started earlier for hydrogen-charged specimens (Table 3), could be fitted well by a linear function σ = *kt*, which meant that the relaxation rate for this part was constant. As for the stage I, the value of |dσ/dt| was the highest for the specimen charged for 100 h, which directly showed the hydrogen-assisted faster dislocation movement/annihilation.

#### 3.1.3. Strain Rate Tests

**Figure 4:**
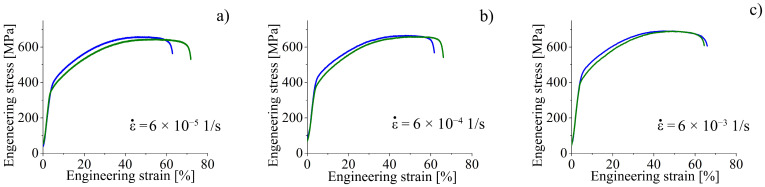
(**a**–**c**) Engineering stress vs. engineering strain for hydrogen-free (green lines) and hydrogen-charged (blue lines) specimens. Strain rates are given on the figures.

The effect of the strain rate on the deformation behavior of the hydrogen-free and hydrogen-charged for 100 h specimens is shown in Figure 4. The data on the mechanical properties of the specimens, calculated using the diagrams in Figure 4, are given in Table 4. The increase in the strain rate is accompanied with the decrease in the total elongation of the specimens and increase in their yield strength and tensile strength (Table 4). The hydrogen-assisted growth in the yield strength of the specimens is observed for all strain rates in the interval 6 × 10^−5^ 1/s–6 × 10^−3^ 1/s. At the same time, the differences in the tensile strength and total elongation of the hydrogen-charged and hydrogen-free specimens become lower with increasing in strain rate (Figure 4, Table 4). The hydrogen embrittlement index K_H_ decreases with increasing strain rate (Table 4).

### 3.2. SEM Analysis

**Figure 5:**
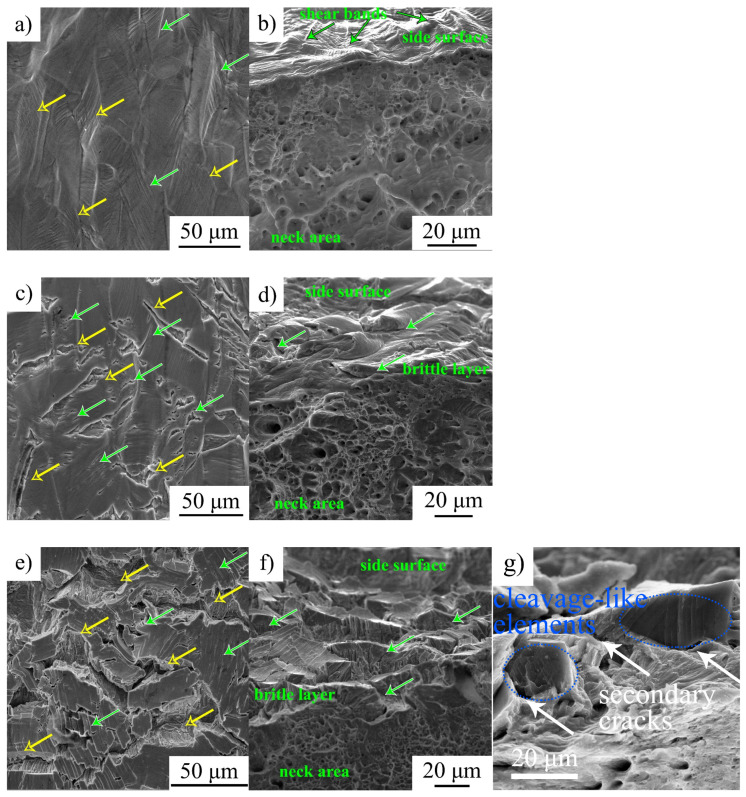
(**a**,**c**,**e**) Side surfaces of the specimens tensile tested to failure; (**b**,**d**,**f**,**g**) fracture surfaces of the specimens: (**a**,**b**) hydrogen-free specimens; (**c**,**d**) hydrogen charging for 8 h; and (**e**–**g**) hydrogen charging for 100 h. Yellow arrows on (**a**,**c**,**e**) show intergranular microcracks, green arrows—transgranular microcracks. Green arrows in (**d**,**f**) show secondary microcracks.

Figure 5 shows side and fracture surfaces of the specimens after deformation with the strain rate of 1 × 10^−4^ s^−1^.

The side surfaces of the hydrogen-free specimens are smooth without any cracks. The traces of the multiple slip are seen inside of grains. The fracture micromechanism of these specimens is a transgranular dimple one (Figure 5b). The side surfaces of hydrogen-free specimens after deformation with different strain rates look similar to that in Figure 5a.

At strain rate ~10^−4^ 1/s, hydrogen charging before tension causes brittle cracking of the specimens’ surfaces during plastic deformation. Both intergranular and transgranular microcracks are seen in Figure 5c,e. Transgranular cracks develop along slip lines, i.e., {111}-type planes in FCC crystal lattice, which testify that hydrogen could influence the deformation mechanism of the steel (slip and twinning) and confirms the hydrogen–dislocation interaction at a strain rate of the order 10^−4^ 1/s. The fracture surfaces of these specimens are characterized by two areas with different fracture micromechanisms. The central part of the specimen’s failures is in ductile regime, which is similar to that in hydrogen-free specimens. The surface brittle layers, with a thickness of approximately 5 μm for 8 h of charging and up to 17 μm for 100 h of charging, have brittle cleavage-like morphology of the fracture surfaces (Figure 5d,f,g). Between these parts with the dimple and cleavage-like morphologies, the flat facets with traces of the deformation are seen (intermediate layer) (Figure 5f).

**Figure 6:**
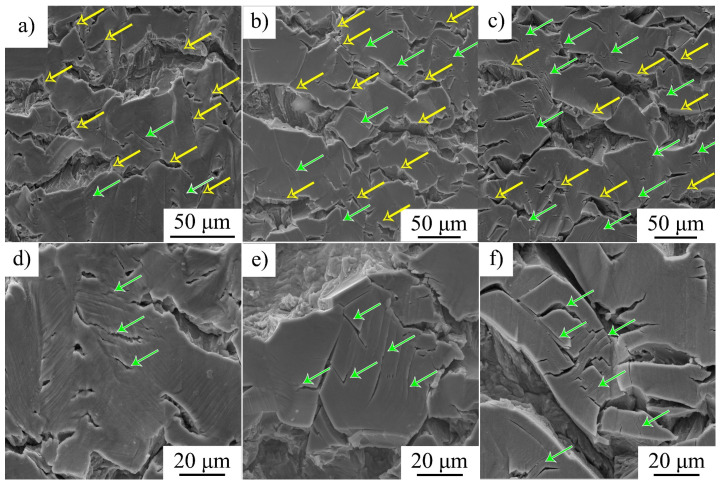
(**a**–**f**) Side surfaces of the hydrogen-charged specimens after tensile tests different strain rates: (**a**,**d**) 6 × 10^−5^ 1/s; (**b**,**e**) 6 × 10^−4^ 1/s; and (**c**,**f**) 6 × 10^−3^ 1/s. Yellow arrows show intergranular microcracks, green arrows—transgranular microcracks.

The number ratio of transgranular and intergranular microcracks on side surfaces of the hydrogen-charged and deformed specimens varies with strain rate. For $\dot{\varepsilon }$ = 6 × 10^−5^ 1/s, intergranular microcracks are predominant and they cause the macroscopic fracture of the specimens. Transtranular microcracks open along slip microbands but their growth is terminated in grain bodies, and they do not develop to the macroscopic cracks (Figure 6a,d). At higher strain rates, the number of parallel transgranular microcracks is much higher (Figure 6b,c,e,f, shown by green arrows). The comparison of the SEM images in Figure 6 shows that (i) the increase in strain rate is accompanied by a coarsening of intergranular microcracks and promotes transgranular cracking, and (ii) at a lower strain rate, slip lines are coarse and numerous relative to those after high strain rate deformation (Figure 6d–f).

**Figure 7:**
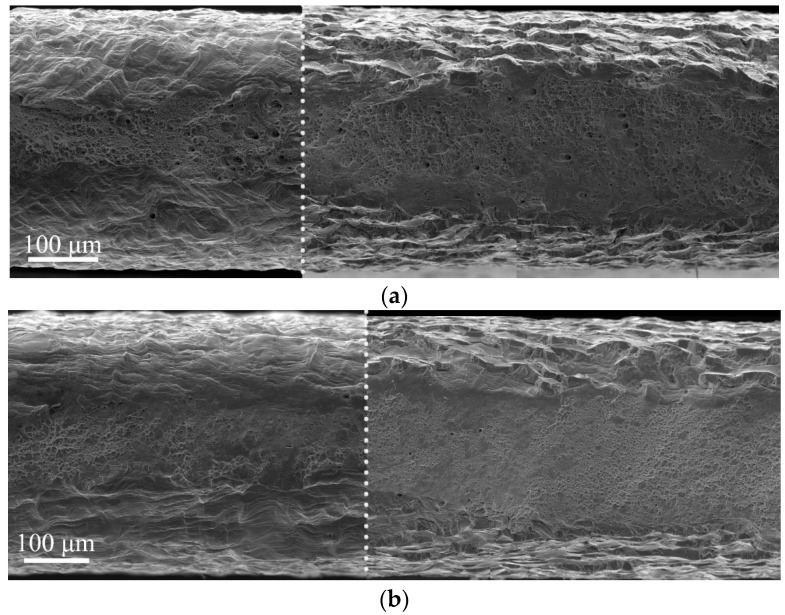
(**a**,**b**) Fracture surfaces of hydrogen-free (to the left from the dotted line) and hydrogen-charged for 100 h (to the right from the dotted line) specimens fractured with different strain rates: (**a**) 6 × 10^−5^ 1/s; (**b**) 6 × 10^−3^ 1/s.

The micromechanisms of the fracture and the thickness of the surface brittle layer in hydrogen-charged specimens do not depend on strain rate, but some details attracted our attention (Figure 7). An intermediate region between brittle surface layers and the ductile matrix looks more ductile for specimens deformed at a higher strain rate (Figure 7). In particular, the dimples in this layer are observed, and they are elongated in one direction at a low strain rate (Figure 7a), but at a high strain rate, a pronounced deformation relief is observed in the intermediate layers (Figure 7b).

### 3.3. TEM Analysis

**Figure 8:**
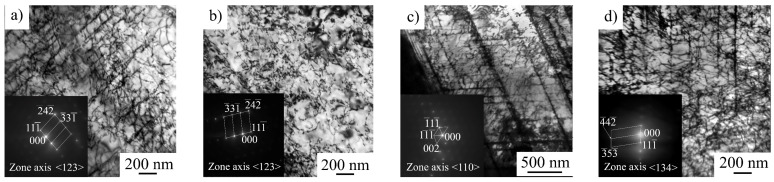
Dislocation arrangement in hydrogen-free (**a**,**b**) and hydrogen-charged (**c**,**d**) specimens before (**a**,**c**) and after stress relaxation (**b**,**d**). Strain rate 10^−4^ 1/s, 10 % strain. The observation area was in the hydrogen-affected surface layer.

Dislocation arrangement of hydrogen-free and hydrogen-charged specimens before (at 10% strain) and after stress relaxation for 60 s are presented in Figure 8. The planar dislocation arrangement is typical of austenitic stainless steels with low stacking fault energy (SFE in 316L steel was measured as 33.6 ± 4.5 mJ/m^2^ [21]). Dislocation arrangement becomes more planar due to the hydrogen saturation (Figure 8a,c). In hydrogen-free specimens, perfect dislocations in several slip systems, stacking faults, and splitted dislocations are seen (Figure 8a). After stress relaxation, the structure of the hydrogen-free sample is recovered (Figure 8b). Dislocation density does not change after stress relaxation (Table 3). 

In hydrogen-charged specimens, perfect dislocations, thin twins and planar dislocation walls along the {111} slip planes are observed (Figure 8c). Hydrogen charging enhances planar slip and assists dislocation accumulation (Table 3). As a result of stress relaxation, the dislocation arrangement becomes more homogeneous; however, the microlocalization of slip is still evident in hydrogen-charged specimens (Figure 8d). The dislocation density in these specimens slightly decreases during stress relaxation (Table 3).

**Figure 9:**
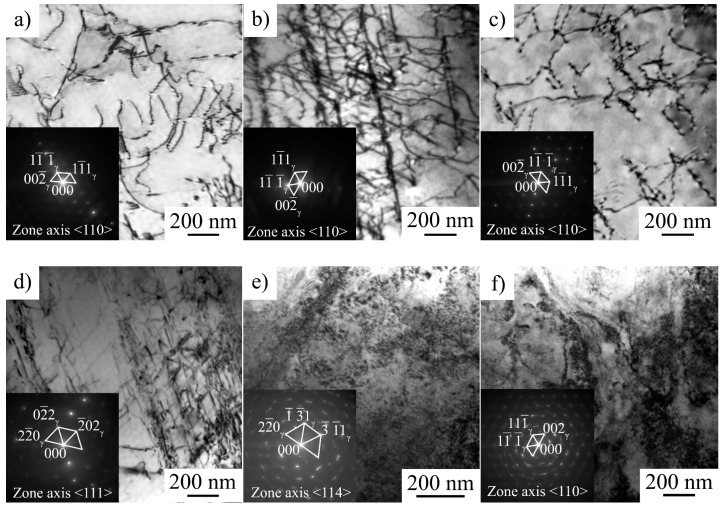
Dislocation arrangement in hydrogen-free (**a**–**c**) and hydrogen-charged (**d**–**f**) specimens at 5% strain: (**a**,**d**) 6 × 10^−5^ 1/s; (**b**,**e**) 6 × 10^−4^ 1/s; and (**c**,**f**) 6 × 10^−3^ 1/s. The observation area was in the hydrogen-affected surface layer.

The microstructure of hydrogen-free specimens is planar independently on strain rate (Figure 9a–c). Dislocation slip is a dominating deformation mechanism and twinning is more active at a higher strain rate, which is typical of austenitic stainless steels [21].

For hydrogen-charged specimens, dislocation arrangement is a strain rate-dependent characteristic (Figure 9e–f). After 5% strain at $\dot{\varepsilon }$ = 6 × 10^−5^ 1/s, the structure of the hydrogen-saturated surface layer is planar (Figure 9d), but at higher strain rates, a very complex dislocation contrast is observed (Figure 9e,f). The dislocation arrangement testifies more of a rather wavy glide than planar one. Dislocation density is as high, as individual dislocations cannot be identified. Numerous extinction contours are observed in the microstructure, which arise due to the hydrogen-affected high internal stresses. The austenitic reflections in the selected area electron diffraction patterns are diffused in azimuth direction (the angle diffusions of the reflections reach 6° and 11° for 6 × 10^−4^ 1/s and 6 × 10^−3^ 1/s, respectively). This also testifies hydrogen-assisted high internal stresses in the microstructure of the hydrogen-saturated layers, which fracture in a cleavage-like mode at strain rates 6 × 10^−4^ 1/s and 6 × 10^−3^ 1/s.

## 4. Discussion

Data on strain rate effect and stress–relaxation tests both testify that hydrogen charging influences dislocation dynamics, microstructure, deformation mechanisms, and fracture micromechanism of austenitic stainless steel AISI 316L.

Hydrogen-assisted increase in the yield strength was previously observed in 300-series steels by D.P. Abraham and C.J. Altstetter [11]. They reported a G/2000 (about 40 MPa) hardening per atomic % of solute hydrogen in 310S stainless steel at $\dot{\varepsilon }$ = 6 × 10^−5^ 1/s. Solid solution strengthening can be associated with a random, ordered, or segregated distribution of the hydrogen in the crystal lattice of the austenitic phase. In our case, the increase in hydrogen charging duration assists solid solution hardening (Table 2), but it can also result from the macroscopically inhomogeneous hydrogen distribution in the specimens and high internal stresses due to the in-depth gradient of the hydrogen concentration (Figure 1). Hydrogen-associated high internal stresses in surface layers are indirectly confirmed by a TEM study of the steel microstructure (Figure 9e,f). High dislocation density and micro stresses form in hydrogen-saturated surface layers during plastic deformation of the specimens in conditions, when dislocation transport of hydrogen into the specimen’s depth is suppressed.

SEM-analysis shows that an increase in strain rate is followed by intensification of transgranular microcracking. Simultaneously, a decrease in the ability of the surface (hydrogen-saturated layer) to deform is observed, the thickness of the intermediate layer declines, and the elongated dimples disappear on their surfaces (Figure 5, Figure 6 and Figure 7). In general, SEM analysis of the side surface shows higher surface cracking at a higher strain rate, but from the point of view of the mechanical properties, steel becomes more resistant to the hydrogen-assisted degradation at high strain rates (Figure 2, Table 2). Similar negative strain rate dependence of the ductility loss was found in [15] for high-carbon steel Fe-33Mn-1.1C and in [11] for 310S stainless steel.

Analysis of the fracture surfaces testify that the hydrogen-affected region is a strain rate-dependent value. For low strain rate 6 × 10^−5^ 1/s, it is much wider than the brittle surface layer identified by the cleavage-like morphology. The elongated dimples in the intermediate layers testify the migration of the hydrogen during tensile deformation in direction to the center of the specimen, and activation of the HELP mechanism [9] of the hydrogen–dislocation interaction there. With increase in strain rate, the speed of dislocations’ movement exceeds the rate of hydrogen diffusion. Therefore, dislocation hydrogen transport is suppressed at the high strain rate 6 × 10^−5^ 1/s. After cracking of the hydrogen-containing layers during plastic deformation, hydrogen weakly influences further deformation and crack growth in intermediate layers. Their deformation and fracture are similar to hydrogen-free material.

As mentioned above, the formation of a highly defective microstructure at high strain rates indicated the presence of a supersaturated solid solution of hydrogen in the surface layer after saturation (Figure 9e,f). At a low strain rate, hydrogen migration with dislocations, HELP mechanism, and stress-assisted diffusion of hydrogen provide gradual hydrogen transport from the surface to the center of the specimens during plastic deformation. Stress relaxation tests directly show the increase in dislocation rate due to the hydrogen–dislocation interaction (Figure 3), which is in the accordance with works [10,22]. This was proven by the growth in the speed of dislocation movement, the planar nature of the dislocation arrangement, and the presence of the microlocalization of slip bands in the surface layers. Hydrogen had no opportunity to diffuse with dislocation cores at a high strain rate. Therefore, hydrogen accumulated near the surface forms a supersaturated solid solution in austenite. In this case, the HELP mechanism does not work. Apparently, the HEDE mechanism [3] is responsible for the generation of transcrystalline cracks at high strain rates.

## 5. Conclusions

Tensile behavior, dislocation arrangement, deformation mechanisms, and fracture of austenitic stainless steel AISI 316L electrochemically charged with hydrogen were investigated at different strain rates in the interval of (10^−5^–10^−3^) 1/s. The main results can be summarized as follows:

(1) The increase in hydrogen charging duration assists solid solution hardening of austenitic phase and provides the increase in the yield strength of the specimens. Along with solid solution hardening, this is associated with the macroscopically inhomogeneous hydrogen distribution in the specimens and high internal stresses due to the in-depth gradient of the hydrogen concentration after charging;

(2) Hydrogen charging facilitates stress relaxation, which is directly related to the dislocation movement and annihilation, enhances planar slip and twinning, and assists dislocation accumulation;

(3) Increase in strain rate inhibits the hydrogen-induced loss of the elongation of the steel specimens due to the suppression of hydrogen transport on dislocations during plastic straining. At a low strain rate (6 × 10^−5^ 1/s), dislocation transport of hydrogen assists HELP-dominated deformation and causes a premature fracture of the specimens. At high strain rates, when hydrogen diffusion with dislocation is terminated, high hydrogen concentrations, internal stresses, and high dislocation density provoke transgranular cracking of the side surfaces of the specimens by the HEDE-dominating mechanism.

## Figures and Tables

**Table 1:** Chemical composition of the steel (mass %), Fe-balanced.

Cr	Ni	Mo	Mn	Si	Ti	C	N	S	P
16.80	13.30	2.70	1.70	0.60	<0.5	0.01	0.08	<0.03	<0.045

**Table 2:** Mechanical properties of hydrogen-free and hydrogen-charged specimens.

Specimen	σ^YS^ [MPa]	σ^UTS^ [MPa]	δ [%]
Hydrogen-free	300 ± 10	970 ± 10	42 ± 3
Hydrogen-charged for 8 h	310 ± 10	970 ± 10	40 ± 3
Hydrogen-charged for 100 h	320 ± 10	930 ± 20	37 ± 4

**Table 3:** Mechanical properties and dislocation density before and after stress–relaxation (ρ_0_ and ρ’, respectively) of hydrogen-charged and hydrogen-free specimens. σ_r_—true stress at start of the relaxation, t_I-II_—time, corresponded to the start of stage II of relaxation (see Figure 3a).

Specimen	σ_r_ [MPa]	t_I-II_ [s]	|dσ/dt|_II_ [MPa/s]	Δσ [MPa]	ρ_0_ [10^9^ m^−2^]	ρ’ [10^9^ m^−2^]
Hydrogen-free	530 ± 20	≈340	0.009 ± 0.002	57 ± 2	7.5 ± 1.0	7.7 ± 2.4
Hydrogen-charged for 8 h	560 ± 20	≈320	0.013 ± 0.002	61 ± 2	12.0 ± 1.8	10.0 ± 0.6
Hydrogen-charged for 100 h	570 ± 20	≈310	0.015 ± 0.002	70 ± 2	–	–

**Table 4:** Mechanical properties (hydrogen-free/hydrogen-charged for 100 h).

Strain Rate, 1/s	σ^YS^ [MPa]	σ^UTS^ [MPa]	δ [%]	K_H_ [%]
6.2 × 10^−5^	360 ± 10/390 ± 10	1050 ± 10/1020 ± 10	54 ± 3/47 ± 4	13
6.2 × 10^−4^	390 ± 10/450 ± 10	1050 ± 20/1030 ± 20	51 ± 3/45 ± 5	12
6.2 × 10^−3^	430 ± 10/470 ± 10	1080 ± 20/1080 ± 20	47 ± 2/44 ± 4	6

## Data Availability

Data available on request.
